# Existing Knowledge of Medication Error Must Be Better Translated Into Improved Patient Safety

**DOI:** 10.3389/fmed.2022.870587

**Published:** 2022-05-17

**Authors:** Craig S. Webster

**Affiliations:** Department of Anaesthesiology and Centre for Medical and Health Sciences Education, School of Medicine, University of Auckland, Auckland, New Zealand

**Keywords:** human factors, systems redesign, medication errors, blame, exhortation, quantitative evidence

## Introduction

The use of medicines in healthcare is safer and more effective than it has ever been, with pharmacovigilance having been active in some form for over 150 years (1). Yet, in 2000, marking what is often thought of as the beginning of the modern patient safety era, two landmark reports appeared, one by the Institute of Medicine in the United States, and the other by the Department of Health in the United Kingdom, demonstrating that avoidable patient harm during healthcare remains extraordinarily expensive and unacceptably common (2, 3). Both reports identified that medication errors are a leading cause of patient harm, and called for a “systems” approach to error prevention. A systems approach promotes the redesign of faulty or error-prone aspects of work systems, environments and procedures, based on an understanding of human factors and ergonomics, rather than exhorting individuals to be more careful or blaming them when patient harm occurs. Employing such a systems approach, some high-profile successes have been achieved in the last 20 years (4, 5). However, medication errors remain a persistent and concerning source of patient harm throughout the world, prompting the World Health Organization (WHO) in 2017 to launch its third Global Patient Safety Challenge, namely Medication Without Harm (6). The WHO Challenge report makes it clear that medication errors typically do not occur because of negligence or carelessness, but because of poor quality or unsafe medication systems, and estimates the global cost of medication errors at $42 billion annually. The report calls for a global reduction in the level of avoidable harm related to medications by 50% over 5 years (6).

### The Persistence of Exhortation, Blame and Trying Harder

The field of human factors has demonstrated that human beings have measurable physical and psychological limits within which individuals operate at their best (7). Once outside of these limits, sheer effort or exhortation to try harder will have little or no lasting effect, and performance will inevitably decline. In a study in the United States based on 5,888 h of direct observation, hospital interns working traditional shifts involving multiple work periods longer than 24 h each month, had a 21% greater chance of making serious medication errors than when working without extended-duration shifts (8). Fatigue levels, such as these, have been equated to blood-alcohol levels in terms of their detrimental effects on performance, suggesting that work shifts of 17 h or more are equivalent to being intoxicated over the legal limit to drive a car (9). No one would accept a clinician practicing while drunk, yet the expectation is often that equivalent fatigue levels should be worked through simply by sheer effort.

A prominent recent reminder that blame is often the first course of action when patient harm occurs can be seen in the response to the tragic death of 6-year old Jack Adcock in 2011 while under the care of Dr. Bawa-Garba (10). Dr. Bawa-Garba did make mistakes during Jack's care, including with medications, but she was operating in a system where any doctor would be have been dangerously overstretched—she was a junior doctor with no senior assistance, in sole charge of six hospital wards, in which the computer system for laboratory results was not functioning at the time (11). Dr. Bawa-Garba was prosecuted for manslaughter, and she and a senior nurse involved in Jack's death, were removed from their professional registers. Such a blame reaction does little or nothing to improve patient safety, as it fails to address the obvious systemic problems underlying the fatal healthcare failure, hence leaving these conditions unaddressed to precipitate further failures in the future. To make matters worse, the legal proceedings against Dr. Bawa-Garba led to widespread concern in the United Kingdom that doctors should no longer record personal reflections on their practice for fear of these records being used against them in a court of law at a later date (12). Given that reflective practice is considered an important part of quality improvement for individual practitioners, it can only be concluded that patient safety in the United Kingdom is now substantially poorer than it was before the Bawa-Garba case. After a long and expensive legal process Dr. Bawa-Garba was reinstated as a medical practitioner in 2018, yet little attention has been given to the system problems that precipitated the tragedy (13). Unfortunately, criminal prosecutions of clinicians for simple human errors are not uncommon, and continue to occur, as demonstrated by the current high-profile case in the United States in which nurse, RaDonda Vaught, has been found guilty of criminally negligent homicide resulting from a medication error (14, 15).

Despite policy level directives that the systems approach should be prioritized over exhortation and blame when aiming to reduce medication errors and patient harm, evidence would suggest that this is often not the case during everyday clinical work (16). For example, in 2020, a 10-year multisite study reported 4223 local corrective measures taken in response to 7072 patient safety incident reports made during perioperative and critical care (17). The study lists the five leading corrective measures as: (1) communication through mortality and morbidity meetings; (2) email alerts; (3) new or revised clinical protocols; (4) a change in material or supplier; and (5) more training. Note that four out of five of these corrective measures are exhortative in nature, and are consistent with the belief that patient safety is best achieved through greater effort on the part of clinicians (e.g., be more aware, follow the new protocol, get better trained). Only one corrective measure (a change in material or supplier), involves a minor change to the work system in which clinicians are expected to perform safely.

## Discussion

### Approaches to the Reduction of Medication Errors That Actually Work

The perioperative period is one of the most intensive phases of medication administration in healthcare, involving some of the most potent agents administered in relatively rapid succession. Anesthesia is widely considered to be a leader in patient safety in healthcare, and in last 20 years the reduction of medication error has been an active area of research in anesthesia (18). In 2021 two systematic reviews of medication safety interventions in anesthesia were published (19, 20), and Table 1 summarizes the primary findings from each of the nine intervention studies included in these two reviews (21–29).

**Table 1:** Intervention studies intended to improve medication error.

**Study**	**Design**	**Intervention**	**Outcome**	**Relative reduction in medication error**
1. Bowdle et al. (22)	Before/after hospital operating room (OR) incident monitoring study	Conventional methods vs. two-stage safety bundle, comprising: colored-coded labels^*^; pre-drawn syringes; removal of high-risk ampoules; smart infusion pumps; barcode-based identity checking and voice confirmation	Baseline: 73 administration errors in 11,709 anesthetic cases Stage 1: 57 administration errors in 14,572 anesthetic cases Stage 2: 56 administration errors in 24,264 anesthetic cases	Stage 1: 37%, *p =* 0.008 Stage 2: further 41%, *p =* 0.005
2. Martin et al. (23)	Before/after hospital OR audit	Anesthesia medication tray reorganization separating frequently-used from high-risk drugs; standardized cart top, medication labeling and color coding^*^	101 labeling errors in 368 syringes vs. 16 in 402	85%, *p < * 0.001
3. Wang et al. (24)	Randomized hospital OR cart reconciliation study	Conventional vs. automated dispensing anesthesia carts with built-in checks	641 errors in 5,394 administrations vs. 396 in 5,418	39%, *p =* 0.001
4. Fawaz et al. (25)	Before/after perioperative observation study in pediatric surgery department	Education on definition and type of medication errors, process of medication use, difference between errors and adverse events, barriers, prevention strategies, and role of clinical pharmacist in the operating room	312 errors in 936 medication orders vs. 224 in 693	3%, *p =* 0.7, ns
5. Bowdle et al. (21)	Before/after hospital OR incident monitoring study	Barcode-based identity check and voice confirmation of medications	59 administration errors in 14,576 cases vs. 55 in 24,276	44%, *p =* 0.002
6. Merry et al. (26)	Randomized, controlled observation study in hospital OR	Barcode-based identity check and voice confirmation of medications; color-coded labels^*^; improved organization of workspace and cart	488 administration and recording errors in 5,084 administrations vs. 471 in 5,680	21%, *p =* 0.045
7. Webster et al. (27)	Controlled hospital OR incident monitoring study	Barcode-based identity check and voice confirmation of medications; color-coded labels^*^; improved organization of workspace and cart	268 administration errors in 550,105 administrations vs. 58 in 183,852	35%, *p =* 0.002
8. Merry et al. (28)	Before/after perioperative hospital incident monitoring study	Conventional methods vs. color-coded medication infusion labels^*^ that included nomogram removing the need for dose calculations, and barcode-based identity check	7 administration errors in 18,491 anesthetic cases vs. 0 in 10,655	100%, *p =* 0.045
9. Fasting et al. (29)	Before/after hospital OR incident monitoring study	Black and white labels vs. color-coded labels^*^ (without barcode identity check) and education on medication error	40 administration errors in 28,971 anesthetic cases vs. 23 in 26,455	37%, *p =* 0.07, ns

* *Color coding according to the international color-code standard for anesthetic labels*.

Seven of the nine studies showed significant reductions in medication errors, demonstrating a median (range) reduction in error of 39% (21–100%)—Table 1. Looking more closely at the details of the studies in Table 1 we can see that the two studies that failed to show a significant reduction in medication error relied wholly or largely on interventions comprised of education (study 4 and 9) (25, 29). Study 9 did include user-applied color-coded labels, but the use of these labels was voluntary, and the labels did not incorporate a bar-code, hence could not allow an automated identity check of a labeled syringe before administration to the patient (29). The seven studies which successfully showed reductions in medication error all used multimodal systems-based approaches combining elements such as: better workspace layout; standardization of medication labels, carts and trays; color-coding; bar-coding; auditory confirmation of medication when label scanned during an automated identity check; and “smart” infusion pumps and dispensing carts, designed to be easier to use and have built in safety checks. Study 8 also included a nomogram incorporated into the medication label to remove the need for dose calculations during its use, hence reducing the cognitive load on the clinician (28). All studies using color-coding did so consistent with the international color-code standard for anesthetic labels, a standard that assigns a color to a particular pharmacological class of medication (e.g., red indicates muscle relaxants, blue indicates opioids and so forth) (30). Such a color-coding scheme reduces the risk of dangerous inter-class medication swaps (27). The reduction of medication errors by a median of 39% for the successful interventions in Table 1 is of the magnitude called for by the WHO Global Patient Safety Challenge (6). However, further work will be needed to achieve a similar level of reduction in patient harm resulting from reduced errors.

Such multimodal approaches are consistent with modern human factors principles in the sense that they provide multiple opportunities to cue correct actions and check actions before a medication is administered, hence trapping, or blocking errors before they reach the patient. For this reason, they have been called strong approaches for reducing errors in complex workplaces, whereas initiatives based only on education or other forms of exhortation have been called weak approaches (31). Education should of course underpin all multifactorial systems-based interventions, but specifically to inform clinicians why a change to their workflow is needed and how the new approach should be carried out correctly. Education is therefore a necessary but insufficient condition for effective error reduction—if the physical work environment does not support and facilitate safer ways of doing things, then it is unlikely that education alone will change long established work habits in the face of time pressures and workload demands (as demonstrated in the results in Table 1). Good systems redesign should make it simultaneously easier and safer for clinicians to do their jobs.

### The Role of Simulation

All studies in Table 1 were based in the clinical environment. However, high-fidelity healthcare simulation has an emerging role in modern systems redesign. Entire work environments, including human-patient mannikins, can be simulated to a level of fidelity that meaningfully engages real teams of expert clinicians (32, 33). This can allow new procedures, techniques, or technology to be tested, practiced and fine-tuned without any risk to real patients. Such simulation can also allow teams to practice their response to rare or dangerous crises situations, so that they are prepared to effectively deal with them if encountered in the real world.

Incident reporting is well established in healthcare, but better use of such incident data could be made in terms of identifying error-prone aspects of work systems where redesign efforts should be targeted (34). Such remedial approaches could then be tested and practiced during simulation before being phased into clinical practice. Such an approach would close the loop by bringing tested safety interventions back to the clinical workplace from which the incidents were reported in the first place. Such a comprehensive safety management system would extend existing incident reporting schemes by better connecting them to active efforts in systems redesign, and would go substantially beyond education, exhortation or blame (35).

## Conclusions

Healthcare practitioners are typically conscientious professionals doing their absolute best. Yet, when medication errors happen or patients are harmed the first reaction of many in healthcare organizations remains a call for more training at best, or blame at worst. This is despite the fact that we know from the field of human factors that exhortation and blame are weak and ineffective approaches to achieving lasting improvements in complex work environments. Strong approaches, such as systems redesign, have been promoted in healthcare through policy documents for at least 20 years. The healthcare literature also contains the results of many successful research studies showing reductions in medication errors and patient harm by employing systems redesign. However, there remains a lack of translation of the existing knowledge on patient safety into the everyday operational decision making and clinical work of many in healthcare throughout the world. To reduce the persistent problem of medication error and improve patient safety, we must consider the larger system in which clinicians work, and consider ways in which cues, checks and other aids can be built into the workplace to support and facilitate clinicians and their good intentions to perform safely. Data from incident reporting schemes can be made better use of to target error-prone aspects of healthcare systems, and simulation can be used to test, refine and practice such interventions before their use on patients—thus creating a more comprehensive safety management system. Education is a necessary but insufficient condition for lasting error reduction. Fatigue is also known to substantially increase rates of medication error and must be managed. Successful intervention studies in anesthesia using systems redesign have shown significant reductions in medication errors of the magnitude called for in the WHO's third Global Patient Safety Challenge. Many of these medication safety interventions can be translated relatively easily into other healthcare domains, including standardization and better layout of workplaces, color-coding, and bar-coding to allow medication identify checks. The technology involved in these safety innovations is not expensive, and is certainly less expensive than continuing to harm patients at current rates.

## Author Contributions

The author confirms being the sole contributor of this work and has approved it for publication.

## Conflict of Interest

CW is a minor shareholder in a company called SAFERsleep LLC, which manufacturers an anesthesia record system.

## Publisher's Note

All claims expressed in this article are solely those of the authors and do not necessarily represent those of their affiliated organizations, or those of the publisher, the editors and the reviewers. Any product that may be evaluated in this article, or claim that may be made by its manufacturer, is not guaranteed or endorsed by the publisher.
